# A Mutation in *Myo15* Leads to Usher-Like Symptoms in LEW/Ztm-*ci2* Rats

**DOI:** 10.1371/journal.pone.0015669

**Published:** 2011-03-29

**Authors:** Nadine Held, Bart M. G. Smits, Roland Gockeln, Stephanie Schubert, Heike Nave, Emily Northrup, Edwin Cuppen, Hans J. Hedrich, Dirk Wedekind

**Affiliations:** 1 Institute of Laboratory Animal Science, Hannover Medical School, Hannover, Germany; 2 Hubrecht Institute, KNAW and University Medical Center Utrecht, Utrecht, The Netherlands; 3 Clinic of Ophthalmology, Hannover Medical School, Hannover, Germany; 4 Institute of Human Genetics, Hannover Medical School, Hannover, Germany; 5 Institute of Functional and Applied Anatomy, Hannover Medical School, Hannover, Germany; Johns Hopkins, United States of America

## Abstract

The LEW/Ztm-*ci2* rat is an animal model for syndromal deafness that arose from a spontaneous mutation. Homozygous animals show locomotor abnormalities like lateralized circling behavior. Additionally, an impaired vision can be observed in some animals through behavioral studies. Syndromal deafness as well as retinal degeneration are features of the Usher syndrome in humans. In the present study, the mutation was identified as a base substitution (T->C) in exon 56 of *Myo15*, leading to an amino acid exchange from leucine (Leu) to proline (Pro) within the carboxy-terminal MyTH4 domain in the proteins' tail region. *Myo15* mRNA was expressed in the retina as demonstrated for the first time with the help of *in-situ* hybridization and PCR. To characterize the visual phenotype, rats were examined by scotopic and photopic electroretinography and, additionally, histological analyses of the retinas were conducted. The complete loss of sight was detected along with a severe degeneration of photoreceptor cells. Interestingly, the manifestation of the disease does not solely depend on the mutation, but also on environmental factors. Since the LEW/Ztm*-ci2* rat features the entire range of symptoms of the human Usher syndrome we think that this strain is an appropriate model for this disease. Our findings display that mutations in binding domains of myosin XV do not only cause non-syndromic hearing loss but can also lead to syndromic disorders including retinal dysfunction.

## Introduction

The LEW/Ztm-*ci2* rat (*ci* = circling), previously described as an animal model for syndromal deafness, arose as a spontaneous mutation in the inbred strain LEW/Ztm in 1991. The mode of inheritance of the deviant phenotype is autosomal recessive. In addition to deafness, the animals show spontaneous lateralized circling behavior, stargazing, locomotor hyperactivity, moderate ataxia and the inability to swim. Motor abnormalities can either be induced through stress or occur spontaneously [1]. The symptoms manifest during the first three weeks of life. Histological analysis of the inner ear revealed that the organ of Corti, which includes inner and outer hair cells, is completely absent or occasionally reduced. However, inner hair cells and outer hair cells of the vestibular organs are both present. Characteristic effects in this area are shortened stereocilia, a lower number of ganglion cells and a reduced thickness of axons [2].

There are a number of mouse and rat strains that show a phenotype similar to the LEW/Ztm-*ci2* rat such as the shaker-2 mouse (STOCK *Myo15^sh2^*/J), which incidentally carries a mutation in the *Myo15* gene [3].

Myosin XV is assigned to a protein family, namely the unconventional myosins, which are involved in various cellular functions such as cell motility, cytoplasmatic transport and movement processes, endocytosis and exocytosis, regulation of ion channels and also play an important role within the actin cytoskeleton [4]–[7]. Function of the myosin XV protein includes the maintenance of actin organization in hair cells of the organ of Corti. Therefore, it seems to be critical for the normal cytoskeletal morphology [3].

In humans, mutations in *Myo15* cause the non-syndromic autosomal recessive profound hearing loss disorder (DFNB3) [8], [9]. Mutations in some of the other unconventional myosins, such as myosin VI and VIIa, have also been reported to be responsible for hereditary deafness in humans, mice and rats [10]–[12], [13].

The shaker-1 (STOCK *Myo7a^sh1^*/J) mouse and the tornado rat (Crl:WI-*Myo7a^tnd^*) are further examples for strains showing phenotypes comparable to LEW/Ztm-*ci2* rats. In both cases the underlying mutations were traced to the *Myo7a* gene [13], [14].

*MYO7A* is one of several genes responsible for the human Usher syndrome (USH) [15]. This is a clinically variable human disease, with an autosomal recessive mode of inheritance, characterized by congenital sensorineural hearing loss, vestibular dysfunctions and visual impairment. The disease is caused by several mutations in proteins integrated in the Usher protein network. The syndrome has three distinct clinical subtypes [16] referred to as USH1, USH2, USH3, which vary in the severity of disease manifestation with USH1 being the most severe form. Patients suffer from profound hearing loss, constant vestibular dysfunction (balance deficiency) and a prepubertal onset of retinitis pigmentosa. The latter is characterized by a progressive degeneration of the retinal photoreceptor cells [17] that leads to night blindness and visual field loss over the course of several decades [18], [19].

We were able to identify the mutation causing the deviant phenotype of the LEW/Ztm-*ci2* rat in the *Myo15* gene. During our study we only observed the retinal phenotype in some animals and believe that it is dependent on environmental factors. Our findings confirm what was already suggested by Gockeln et al., 2003 and Löscher et al., 2009/2010 [20]–[22]: The LEW/Ztm-*ci2* rat is a valuable animal model for the human Usher syndrome.

## Materials and Methods

### Animals

Homozygous LEW/Ztm-*ci2* rats of both genders were used and all animals originated from the CAF breeding colony. The coisogenic background strain LEW/Ztm (F122/123) served as control. Age of animals used for electrophysiological or histological investigations ranged between 400 and 750 days, with an average of 550 days.

Husbandry and experiments were in accordance with the German Animal Welfare Legislation (*Tierschutzgesetz*: http://bundesrecht.juris.de/bundesrecht/tierschutzg/) and AALAS (http://foundation.aalas.org/). All experiments were also approved by the local Institutional Animal Care and Research Advisory Commitee and by the local government, namely the Lower Saxony State Office for Consumer Protection and Food Safety (approval ID: 509c-42502-03/651).

### Husbandry

Animals were kept in a controlled environment with a temperature of 22±2°C, relative humidity of approximately 55%, and artificial light from 5∶30 h to 19∶30 h. The rats were kept on a sterilized commercial softwood granulate bedding (Lignocel, Altromin; Lage, Germany) and received commercial pellet diet (Altromin, 1314) and water *ad libitum*. Rats were kept alone, as pairs, or as sibling groups in wire-topped type III Macrolon® cages (Techniplast, Italy) in Hannover Medical School. In the University of Veterinary Medicine Hannover animals were kept alone. Microbiological status was monitored according to the FELASA recommendations [23]. In both facilities mutant (LEW/Ztm-*ci2*) and control (LEW/Ztm) strains were housed in the same rooms under the same conditions.

### DNA isolation

For tissue collection, animals were sacrificed by cervical dislocation after anesthesia with CO_2_. Lysis of tissue samples (ear, tail) was prepared overnight at 55°C in 400 µl lysis buffer, containing 100 mM Tris (pH 8.5), 200 mM of NaCl, 0.2% of SDS, 5 mM of EDTA, and 100 µg/ml of freshly added Proteinase K. Samples were centrifuged for 15 min at 6000 g and the supernatant was transferred to a fresh tube or plate. Genomic DNA was isolated by adding an equal amount of isopropanol, mixing and subsequent centrifugation for 20 min at 6000 g. Pellets were rinsed with 70% ethanol and dissolved in 400 µl H_2_O. For PCR, 5 µl of a 50 fold dilution in water was used.

### PCR and sequencing

Resequencing of all exons of *Myo15* and of *Kcnj12* was performed using LIMSTILL, LIMS for Induced Mutations by Sequencing and TILLing (http://limstill.niob.knaw.nl). LIMSTILL was used to generate the *Myo15* project and visualize the gene structure based on Ensembl file ENSRNOG00000028597. The primer design application within LIMSTILL is Primer3-based and parameters are set to design primers with an optimal melting temperature of 58°C.

PCR was performed using a touchdown thermocycling program (92°C for 60 sec; 12 cycles of 92°C for 20 sec, 65°C for 20 sec with a decrement of 0.4°C per cycle, 72°C for 30 sec; followed by 20 cycles of 92°C for 20 sec, 58°C for 20 sec and 72°C for 30 sec; 72°C for 180 sec; GeneAmp9700, Applied Biosystems). PCR reaction mixes contained 5 µl genomic DNA, 0.2 µM forward primer and 0.2 µM reverse primer, 200 µM of each dNTP, 25 mM Tricine, 7.0% Glycerol (w/v), 1.6% DMSO (w/v), 2 mM MgCl_2_, 85 mM Ammonium acetate pH 8.7 and 0.2 U Taq Polymerase in a total volume of 10 µl.

PCR products were diluted with 20 µl water and 1 µl was used as template for the sequencing reactions. Sequencing reactions, containing 0.25 µl BigDYE (v1.1; Applied Biosystems, Nieuwerkerk a/d IJssel, The Netherlands), 3.75 µl 2.5× dilution buffer (Applied Biosystems) and 0.4 µM gene specific primer in a total volume of 10 µl, were performed using cycling conditions recommended by the manufacturer. Sequencing products were purified by ethanol precipitation in the presence of 40 mM sodium-acetate and analyzed on a 96-capillary 3730XL DNA analyzer (Applied Biosystems). Sequences were analyzed using PolyPhred [24].

### Real Time PCR

RNA was isolated using the RNeasy Lipid Tissue Mini Kit (Qiagen) according to manufacturers' instructions. Pituitary gland tissue from LEW/Ztm and LEW/Ztm-*ci2* rats as well as retina tissue collected from LEW/Ztm rats were disrupted and homogenized in QIAzol Lysis Reagent. Then, from 1 µg of RNA, cDNA was synthesized using the Omniscript Reverse Transkription Kit (Qiagen) according to the manufacturer's recommendations. Primers for the *Myo15* fragment were designed using the Oligo 6 program (Molecular Biology Insights); primer: *Myo15*-mRNA fw 5′>ACCTGCCCAGTGTGCGTGAG<3′, *Myo15*-mRNA rev 5′>TGGTGGGGACTGAG TGCCTG<3′. Reaction mixes contained 10 ng cDNA, 2,5 µl biotherm Buffer (with 15 mmol MgCl), 1,5 µl of each primer (10 µM), 0,5 µl dNTP's (10 mM), 2 µl SYBR® Green, 1 U Biotherm Taq in a total volume of 25 µl. Reaction was done using the opticon cycler (MJ Research) with the following cycling conditions: 95°C for 3 minutes, 40 cycles of 95°C for 30 sec., 67,7°C for 30 sec. and 72°C for 30 sec. followed by 72°C for 10 min. Amplified DNA fragments were separated by electrophoresis using 3∶1 Biozym Sieve agarose® (Biozym).

### Electroretinography (ERG)

All procedures involving animals adhered to the Association for Research in Vision and Ophthalmology statement for the use of animals in ophthalmic and vision research. Electroretinograms were recorded according to the standard of the International Society for Clinical Electrophysiology of Vision [25]. The ERG equipment comprised a Ganzfeld bowl, a DC amplifier and a PC-based control and recording unit (Roland Consult, Brandenburg/Wiesbaden, Germany).

Rats were dark-adapted overnight (at least 12 hours). Pupils were dilated using 2,5% phenylephrine and 1% tropicamide, which was administered 20 minutes before measurement. Rats were anesthetized with a mixture of Ketaminhydrochloride (100 mg/kg BW i.p.) and Xylazine (4 mg/kg BW i.p.). Needle electrodes, subcutaneously placed, served as reference (ear base) and ground (tail base) electrodes.

After topical anesthesia of the eyes with metacaine, ca. 0,5 mm circular gold-wire loop electrodes were placed over the bulbi. All procedures were carried out in absolute darkness using red light. Scotopic (rod) recordings were performed with a series of white single flashes with six increasing stimulus intensities reaching from 0.01 to 30.0 cds/m^2^. On each level 3 to 5 flashes were recorded with a frequency of 0,2 to 0,1 Hz and averaged. Interstimulus intervals between levels lasted 30 sec. In a second step, rats were light adapted for 10 minutes with a background of 25 cd/m^2^. Photopic (cone) responses were recorded with a series of five increasing stimulus intensities of 3.0 cds/m^2^ up to 100 cds/m^2^. The a-wave amplitude was measured from the prestimulus baseline to the minimum value of the first negative deflection. The b-wave values were measured from the trough of the a-wave to the maximum positive value.

### Histology

For histological evaluations, eyes were fixed in glutaraldehyde. After dehydration in a graded ethanol series (10%, 30%, 50%, 70%, 80%, 90%, 95% and absolute ethanol), the tissues were embedded in Technovit 7100 (Heraeus Kulzer, Wehrheim, Germany) according to the manufacturer's recommendation. After infiltration with infiltration solution (hardener 1 and Technovit 7100) for 12 h, the eyes were cut into halves along the line of the optic nerve and embedded in Histoform S according to the manufacturer's recommendation. Microtomy was executed strictly perpendicular to the retinal surface. Serial sections of 1–3 mm were stained with haematoxylin (Gill) and eosin.

### Comparison of environmental factors

Microbiological monitoring was performed according to FELASA recommendations [23]. Light intensity was measured in different locations in the room and in three different rack levels (top row, bottom row and middle row) in front of the cages and inside the cages. For analysis an illuminance meter (roline digital luxmeter) was used.

### *In situ* hybridization *Myo15* mRNA

Eyes were postfixed in 4% paraformaldehyde for 24 h at 4°C and embedded in paraffin. The following antisense oligonucleotide probe was used: 5′-CATCGTCATCCGCTTCCCCGGACGACGCGG-3′. The 5′-end digoxigenin-labeled probe recognizes *Myo15* and was obtained from MWG-Biotech AG (Ebersberg, Gemany). A corresponding sense oligo-probe was used as control. Deparaffinized, lightly dried 4 µm sections were rinsed in TBS, 200 mM HCl, and 0.5% acetic anhydride for 20 min, followed by a digestion with 0.1% proteinase K for 30 min at 37°C. Slides were rinsed again in TBS, dehydrated in ascendant concentrations of ethanol, and stretched at 95°C for 5 min. Sections were hybridized at 45°C for 18 h, followed by washings with 2×SSC (standard saline citrate) for 3×20 min, 50% formamide (in SSC) for 3×20 min at 55°C and 1×SSC for 2×15 min. Afterwards slides were incubated with anti-DIG AP for 30 min and NBT/BCIP for 2 h (DIG Nucleic Acid Detection Kit, Roche Diagnostics GmbH, Mannheim, Germany). Slides were viewed with an Axioplan 2 imaging microscope (Zeiss, Jena, Germany) for *in situ* mRNA expression.

## Results

### The mutation of the LEW/Ztm-*ci2* rat was traced to a base substitution in exon 56 of *Myo15*

In a previous study a genome-wide scan of a (LEW/Ztm-*ci2*×BN/Ztm) F1×LEW/Ztm-*ci2* backcross population demonstrated a strong association of the circling 2 phenotype with a region localized centrally in RNO10. Rat BAC clones were used to create a physical map of this region of interest. Two candidate genes, *Myo15* and *Kcnj12*, were selected based on their genomic localization and involvement in a similar pathology in human and mouse [26].

**Figure 1:**
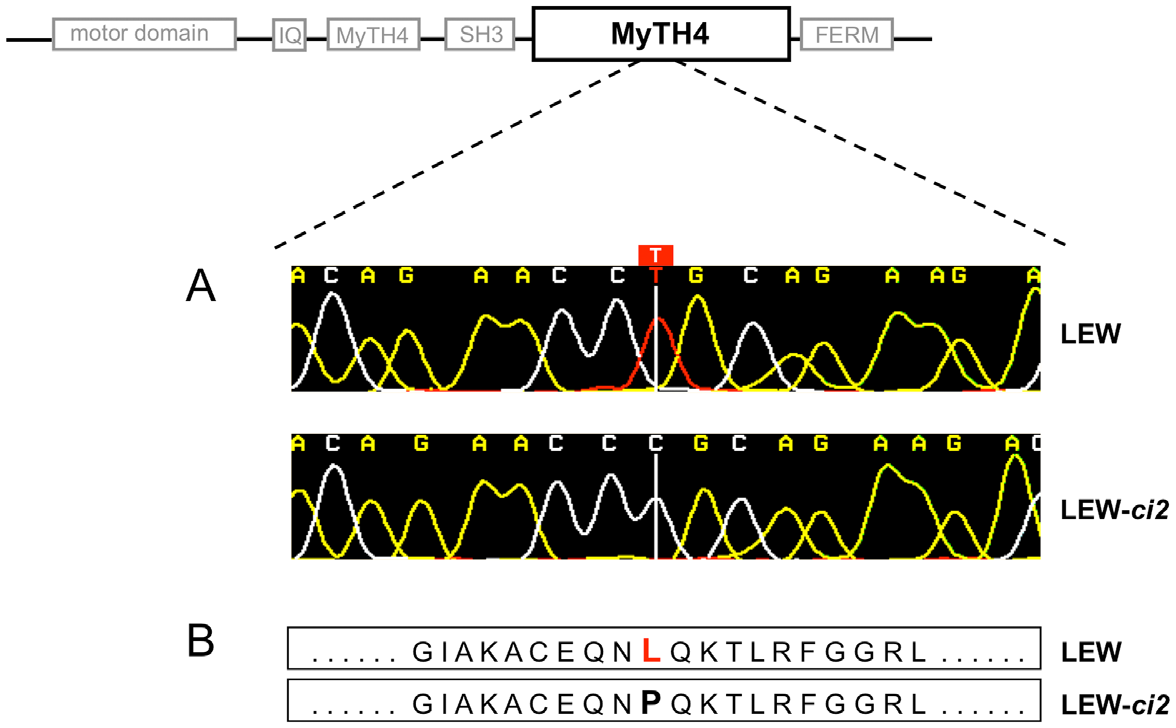
Illustration of the LEW/Ztm-*ci2* mutation in the MyTH4 domain of the *Myo15* gene. (**A**) Polyphred view of the single point mutation found in exon 56 in the C-terminal MyTH4-domain. (**B**) The base substitution (T→C) does not cause a stop codon but leads to an exchange of the amino acid Leucin for Proline at position 3157 in the Myosin XV protein.

To identify the mutation we resequenced exons of both genes using PCR and conventional dideoxy sequencing. Two non-reference alleles of *Myo15* were identified by comparing sequences of the mutated rat strain LEW/Ztm-*ci2* and its co-isogenic background strain LEW/Ztm. The detected base substitution from T to C at position 43612 in exon 56 leads to an amino acid change from leucine to proline (L3157P) within the carboxy-terminal MyTH4 (myosin tail homology-4) domain of the protein (Fig. 1). The leucine residue at position 3157 is conserved throughout the vertebrate lineage, according to MultiZ alignments in the UCSC Genome Browser (http://ucsc.genome.gov). The PolyPhen program predicted this amino acid change to be “possibly damaging” to protein function. Resequencing of *Kcnj12* revealed no additional mutation.

### Expression of *Myo15* transcripts

To determine whether the base pair substitution affects *Myo15* mRNA expression in general, a Real Time PCR of a *Myo15* specific fragment was carried out. RNA was extracted from the pituitary gland of LEW/Ztm-*ci2* and LEW/Ztm rats as the highest *Myo15* RNA expression has been reported for this tissue [27]. *Myo15* signals were normalized against beta-actin signals. The results indicate that there are no significant differences in *Myo15* transcript levels in the pituitary glands of the LEW/Ztm-*ci2* rat compared to rats of the LEW/Ztm background strain.

### Electroretinographic investigations indicated an involvement of the visual system

**Figure 2:**
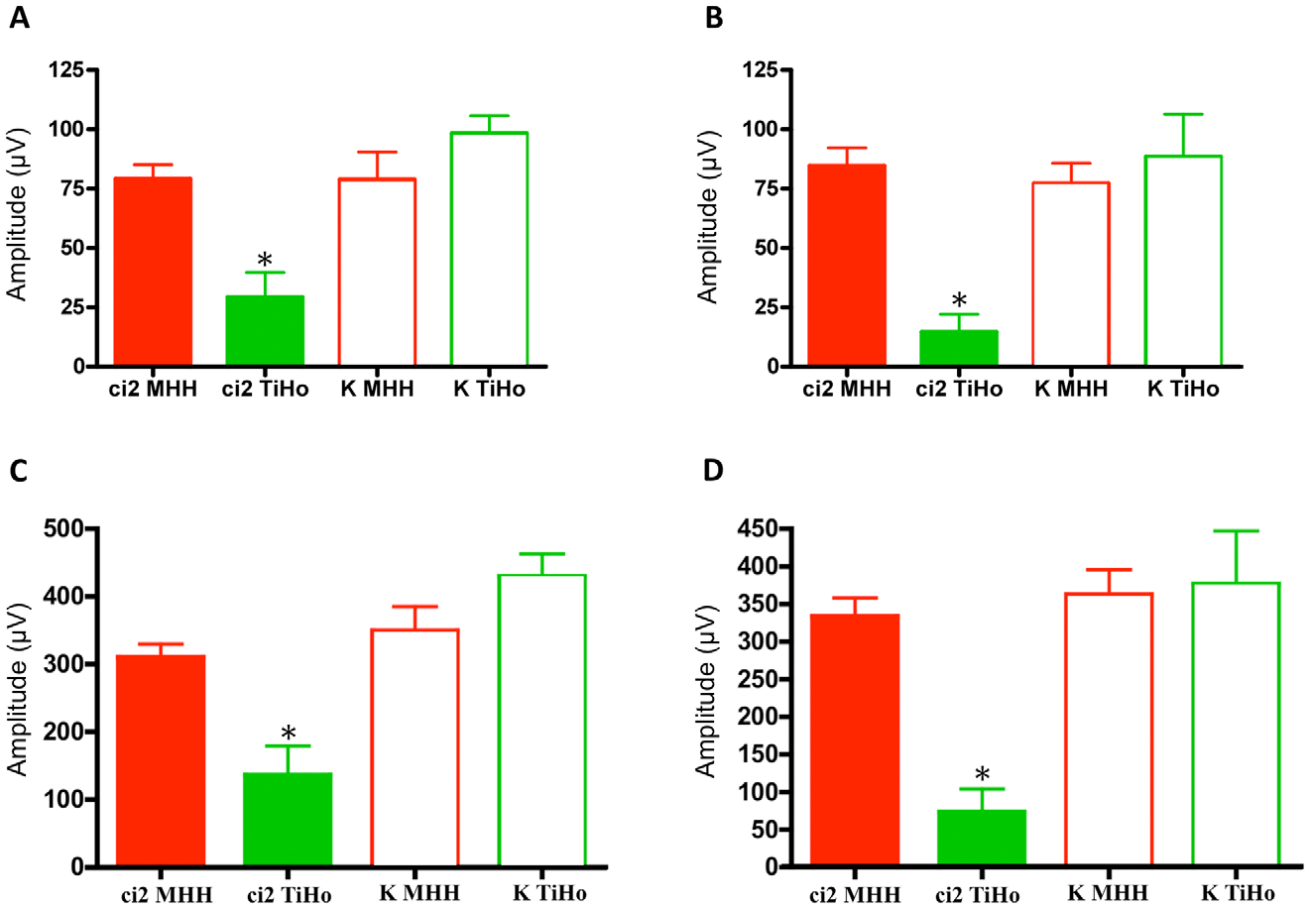
Mean values of rod responses recorded by electroretinography. Average skotopic a- and b-wave maximum amplitudes elicited by a 3 cds/m^2^ light stimulus. (**A**) a-wave amplitudes of the left eyes of LEW/Ztm-*ci2* (ci2) rats from two different hygienic units, MedSchool (MHH) and VetSchool (TiHo) and corresponding controls (K) (**B**) a-wave amplitudes of the right eyes (**C**) b-wave amplitudes of the left eyes (**D**) b-wave amplitudes of the right eyes; responses of *ci2* mutants of the VetSchool group are strongly increased compared to controls and mutant rats of the MedSchool group *significant differences compared to controls p<0.05 are indicated with an asterix.

Behavioral observations of some LEW/Ztm-*ci2* rats using several screening techniques for sensory functions [28], [29] and preliminary electrophysiological investigations indicated that the vision might be impaired (pers. comm., v. Hörsten, data not shown) [20]. To characterize the eye phenotype of this strain, an electroretinographic survey of LEW/Ztm-*ci2* rats (n = 7 per group) and LEW/Ztm rats (n = 7 per group) was carried out. Owing to its results, animals were subdivided into two groups. One group included only animals that were born, raised and maintained at the Central Animal Facility of Hannover Medical School (group MedSchool) for their entire life. In this group, the electrophysiological examinations of LEW/Ztm-*ci2* rats and LEW/Ztm control rats solely revealed physiological responses (Fig. 2, 3-A/C).

**Figure 3:**
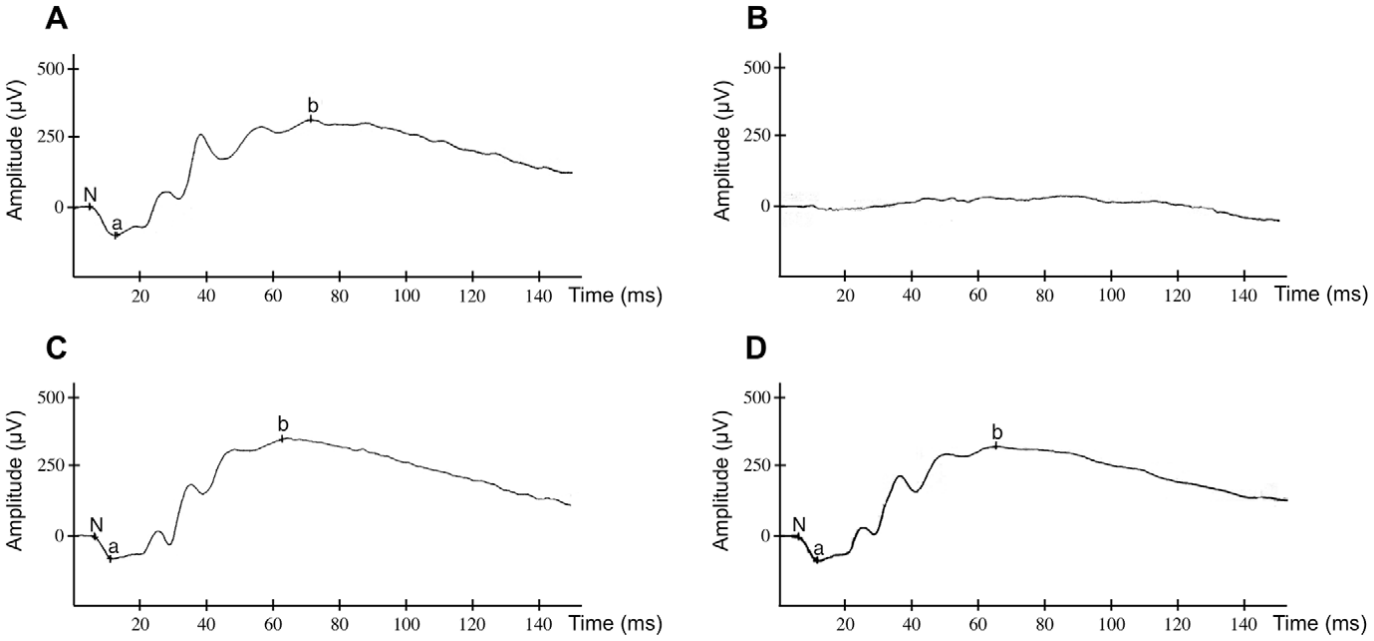
Scotopic electroretinography (ERG) recordings. Electroretinographic responses in (**A**) an unaffected LEW-*ci2* rat (MedSchool), (**B**) an affected LEW-*ci2* rat (VetSchool) with extinct curve (no response), (**C/D**) corresponding, unaffected controls (MedSchool/VetSchool), exemplary for the whole groups. Recorded by a stimulus with a light intensity of 3 cd s/m^2^. N = basic value, a = a-wave, b = b-wave, Time = implicit time.

Animals of the second group were also born and raised in the Central Animal Facility of Hannover Medical School, however, they were at some point transferred and then housed in the University of Veterinary Medicine Hannover (group VetSchool). All LEW/Ztm-*ci2* rats showed decreased responses of the photoreceptor cells, both rods and cones (Fig. 2, 3, Table 1), which means that the incidence of the retinal phenotype was 100 percent. A complete loss of responses was observed in 36% (Fig. 3-B). In contrast, electroretinograms of LEW/Ztm control rats appeared normal (Fig. 2, 3-D, Table 1).

**Table 1:** Retinal phenotypes detected by electroretinography.

Rats	VetSchoolgroup(LEW/Ztm-*ci2*)	VetSchoolcontrols(LEW/Ztm)	MedSchoolgroup(LEW/Ztm-*ci2*)	MedSchool controls(LEW/Ztm)
1	0	UA	UA	UA
2	0	UA	UA	UA
3	0*A	UA	UA	UA
4	A	UA	UA	UA
5	A	UA	UA	UA
6	A	UA	UA	UA
7	A	UA	UA	UA

0 = no response, animal blind; A = significantly reduced response, animal affected; UA = normal response, animal unaffected.

*extincted ERG amplitudes recorded in just one eye of that animal, amplitudes of the second eye significantly decreased.

### Retinal degeneration was discovered by histological examination

**Figure 4:**
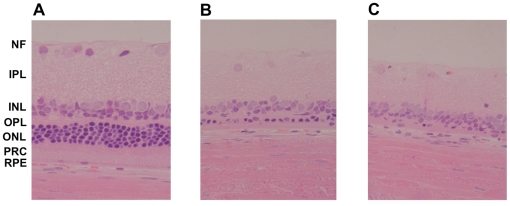
Histological examinations of retinae from unaffected and affected LEW/Ztm-*ci2* rats. (**A**.) Unaffected LEW/Ztm-*ci2* rat (MedSchool group) showing slight reduction of photoreceptor cells due to age (**B**.) Affected LEW/Ztm-*ci2* rat (VetSchool group) with distinct reduction of number and density of rod and cone somata within the ONL (**C**.) Severely affected LEW/Ztm-*ci2* rat (VetSchool group) cell bodies, inner and outer segments of photoreceptor cells are nearly absent while the remaining retinal layers are arranged in a normal pattern (×400) NF = Nerve fibres, IPL = Inner Plexiform Layer, INL = Inner Nuclear Layer, OPL = Outer Plexiform Layer, ONL = Outer Nuclear Layer, PRC = Inner and Outer Segments of Photoreceptor Cells, RPE = Retinal Pigment Epithelium.

Electroretinography is an appropriate method for the early detection of retinal alterations as morphological changes usually do not appear until ERG responses are dramatically reduced. Due to the strong reduction of photoreceptor responses in LEW/Ztm-*ci2* animals of the VetSchool group we examined the eyes histologically. Three eyes of LEW/Ztm-*ci2* rats and LEW/Ztm rats were looked at per group (MedSchool, VetSchool), 12 samples in total. All samples in LEW/Ztm-*ci2* rats from the VetSchool group showed some degree of pathological alteration ranging from a diminution to a complete loss of the photoreceptor cell layer and the outer nuclear layer (Fig. 4-B/C). Interestingly, the remaining cell layers (inner nuclear layer, inner and outer plexiform layers) and the retinal pigment epithelium appeared intact. In contrast, none of the LEW/Ztm-*ci2* and LEW/Ztm rats of the MedSchool group showed any signs of photoreceptor degeneration (Fig. 4-A).

### *Myo15* transcripts were detected in retinas by *in situ* hybridisation and by Real Time PCR

**Figure 5:**
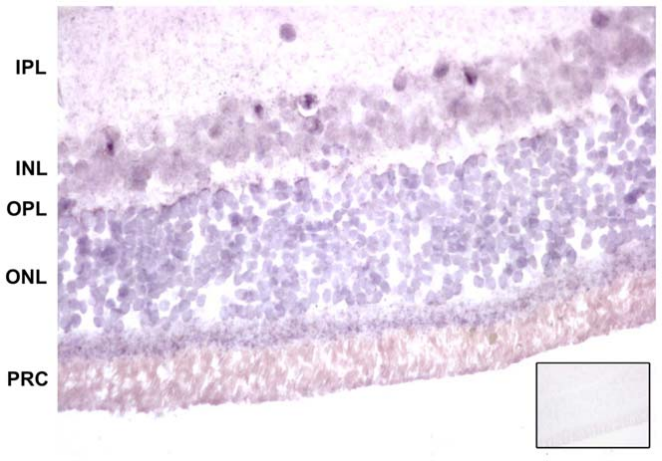
In situ hybridization in a paraffin section of a LEW/Ztm-*ci2* rat indicates the presence of *Myo15* mRNA in the retina. The insert demonstrates the same retina with the sense-control. (×80) IPL = Inner Plexiform Layer, INL = Inner Nuclear Layer, OPL = Outer Plexiform Layer, ONL = Outer Nuclear Layer, PRC = Inner and Outer Segments of Photoreceptor Cells.

Retina sections of affected and unaffected LEW/Ztm-*ci2* animals were hybridized with a 5′-end digoxigenin-labeled probe that recognizes the *Myo15* mRNA in a region not harboring the mutation. *Myo15* transcripts were detected in the retinas of affected (Fig. 5) and unaffected animals. Probe binding was discovered in all retinal layers but to a higher degree in the Outer Plexiform Layer and in the Inner Photoreceptor Cell Layer.

**Figure 6:**
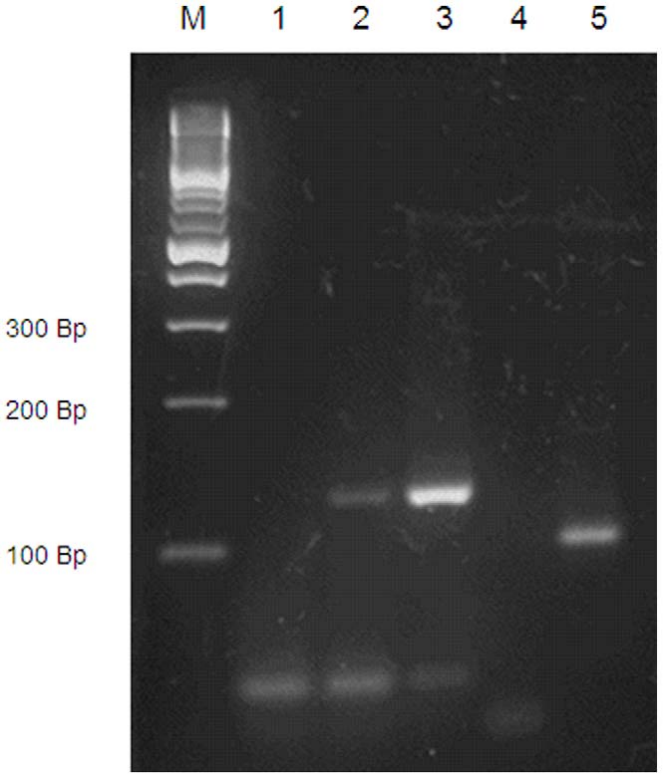
Evidence of *Myo15* expression in the rat retina. Agarose gel results of PCR products obtained from retinas of LEW/Ztm rats. **Lane M** contains the DNA ladder; **lane 1** contains the negative control reaction (no cDNA template, *Myo15* primers); **lane 2** contains cDNA (10 ng) obtained from the retina (*Myo15* primers, expected length of the fragment 125 bp); **lane 3** contains positive control, cDNA (10 ng) obtained from the pituitary gland (*Myo15* primers, expected length of the fragment 125 bp); **lane 4** contains negative control reaction (no cDNA template, *ß-Actin* primers); **lane 5** contains cDNA obtained from the retina (*ß-Actin* primers, expected length of fragment 101 bp).

*Myo15* mRNA was detected in retinas of LEW/Ztm rats by PCR techniques (Fig. 6).

### Differences in environmental conditions of both facilities (MedSchool/VetSchool) did not seem to disturb the vision

LEW/Ztm-*ci2* rats from two facilities display diverging eye-phenotypes. As all animals originate from the same stock of an inbred strain the environmental conditions must be the cause for these disparities. Environmental factors certainly have the potential to cause retinal damage. Excessive light exposure, for example, can cause severe retinal degeneration, particularly in albino rats [30], [31]. The light intensity as well as the duration of light (enlarged light cycle) play a decisive role.

Since unaffected control animals were housed along with affected LEW/Ztm-*ci2* rats, the environmental factors alone cannot be responsible for the retinal phenotype of LEW/Ztm-*ci2* rats. Nevertheless, all factors that have the potential to harm retinal structures were investigated in both facilities.

Microbiological monitoring did not reveal an infection with common rat pathogens except for *Staph. aureus*, *Pasteurella pneumotropica*, *Helicobacter sp.* (MedSchool and VetSchool); *Corynebacterium sp.*, *Rat Parvo Virus* (MedSchool); and *Oxyura syphacica muris* (VetSchool). Borna disease virus wasn't detected in either of the faculties. Furthermore, previous or acute infections were excluded by histological examination of the retinas and by blood tests.

The light intensity measured in the middle of the animal room, in front of the cages and inside the cages, was for the most part comparable between facilities (Table 2). However, the room of the VetSchool was illuminated in a very uneven way. The light intensity inside all cages ranged from 2 to 28 lux, however, 130 lux were observed in the top row of the female's rack in the room of the VetSchool.

**Table 2:** Light intensities inside cages measured at 3 levels of the rack.

measuring point	VetSchool		MedSchool
	females	males	females/males
top level	130 lux	28 lux	6 lux
middle	10 lux	12 lux	6 lux
bottom level	10 lux	6 lux	2 lux

Additional environmental factors (such as diet, water supply, bedding, light cycle etc.) did not differ between both facilities.

## Discussion

In this study we describe the first rat strain carrying a mutation in *Myo15*. Phenotypic features of homozygous LEW/Ztm-*ci2* animals are deafness and spontaneous lateralized circling behavior combined with further motor abnormalities such as stargazing, locomotor hyperactivity and ataxia [1]. Previously, histological investigations were able to show that hair cells of the inner ear and the vestibular organs are damaged or lacking [2]. The mutation has been mapped to a region on rat chromosome 10, including strong candidate genes *Myo15* and *Kcnj12*.

Resequencing of these candidate genes revealed a base substitution (T→C) in exon 56 of *Myo15* causing an amino acid exchange in the C-terminal MyTH4 domain of myosin XV. *Myo15* transcript levels are comparable between mutant and wild-type animals, indicating that the protein function of myosin XV must be altered. According to the recommendations of the rat genome and nomenclature committee this model is now denominated LEW/Ztm-*Myo15^ci2^* rat.

Recessive mutations in *MYO15A* are associated with the profound nonsyndromic hearing loss disorder (DFNB3) in humans [8], [9]. Previously described mutant allels of *MYO15A* are distributed across the length of the gene. A number of DFNB3 causing mutations have been identified and are mainly located in the motor region [8], [9] but also in the N-terminal MyTH4 and FERM domains of the protein's tail [32], [33]. Only recently a missense mutation (L3160F) in the C-terminal MyTH4 domain of myosin XV was detected in a Pakistani family. Although this mutation is located close to the circling-2 mutation the only symptom reported in humans is deafness [32].

The tail domains of myosin XV have several binding sites that interact with other proteins. The co-localization of the myosin XV and whirlin proteins is thought to be a key event for stereocilia elongation [34], [35]. The PDZ domain of whirlin binds to the C-terminal MyTH4-FERM region of myosin XV [34]. As this is the same myosin XV region where the circling-2 mutation occurred, we think it is possible that the binding site is affected.

The mutation in the third PDZ domain of whirlin in humans causes the sensorineural deafness disorder (*DFNB31*). A homologous mutation in the whirlin gene (*Whrn*) in a mouse strain results in a phenotype very similar to that of the shaker-2 mice and circling-2 rats [36]–[39].

Whirlin is part of a protein network performing several functions in neurosensory cells of the retina and the cochlea. It links proteins that are involved in the Usher syndrome, such as myosin VIIa, harmonin b, cadherin-23, protocadherin-15 and VLGR1b [40]. This Usher interactome participates in common pathways of the inner ear and the retina that are disrupted in the Usher syndrome [41]. However, the biochemical and structural mechanism governing the Usher protein complex formation remains largely unclear.

Several rodent strains have been suggested as animal models for the human Usher syndrome (Subtype 1B), for example the shaker-1 mouse (STOCK *Myo7a^sh1^*/J) and the tornado rat (Crl:WI-*Myo7a^tnd^*), respectively. Both animal models show circling behavior, vestibular dysfunctions and profound deafness. However, blindness or progressive loss of vision and degeneration of the retina, as can be seen in USH1-patients, have not been described.

So far, an involvement of myosin XV in the human Usher syndrome (USH) has not been established. However, myosin XV interacts with whirlin - a protein known to be involved in the Usher syndrome (USH2D). Whirlin plays a central role in the Usher protein network, which consists of at least nine different proteins [41], [42]. Since myosin XV is responsible for the transportation of whirlin in the inner ear, a similar function might be imaginable in the retina. To investigate the expression of myosin XV mRNA in the eye, a Real Time PCR and an *in-situ* hybridization were performed. Being able to demonstrate the expression of *Myo15* mRNA in rat eyes for the first time we went ahead and examined whether the LEW/Ztm-*Myo15^ci2^* rat exhibits a retinal phenotype.

Previous studies indicated that the visual system is affected in some of the LEW/Ztm-*Myo15^ci2^* rats (pers. comm., v. Hörsten). Electroretinographical and histological investigations revealed that the loss of function is caused by the degeneration of the retina (Fig. 2, 3). We hypothesize that the mutant form of myosin XV in LEW/Ztm-*Myo15^ci2^* rats is not able to bind to its partner proteins (e.g. whirlin) in the retina and thereby reduces their functions.

Interestingly, LEW/Ztm-*Myo15^ci2^* rats could be subdivided into two groups depending on housing: one susceptible to retinal damage and the other resistant.

In the group housed in the VetSchool the incidence of a retinal phenotype was 100 percent. Histological evaluation revealed a degeneration of the retinas in the affected eyes. The RPE and the inner retina seemed to be totally unaffected, while the outer retina was almost completely disintegrated with only a few photoreceptor cell somata remaining. These findings are very similar to those revealed in a mouse model for Usher syndrome type 2A, described by Liu et al. 2007 [43]. In this mouse model, reduced ERG amplitudes and retinal degeneration were detected solely in aged animals. This finding has also been demonstrated in our LEW/Ztm-*Myo15^ci2^*.

Surprisingly, none of the LEW/Ztm-*Myo15^ci2^* animals of the MedSchool group showed any signs of impaired vision. Therefore, the question arose: Which factors could be responsible for inducing the retinal phenotype in LEW/Ztm-*Myo15^ci2^* rats?

A new genetic defect can be excluded since all animals were born in the same breeding colony in the Central Animal Facility of Hannover Medical School and some even stem from the same breeding pair. The only difference between these animals was their environment. Since control animals, which were kept under the same environmental conditions did not show any signs of retinal impairment, the environmental factors alone could not be held responsible for the retinal phenotype in LEW/Ztm-*Myo15^ci2^* rats. Comparison of environmental conditions revealed some differences between the two facilities but so far it has not been possible to pinpoint to any obvious factors affecting the animals' sight. Involvement of certain pathogens, e.g. coronavirus or Borna disease virus, has been reported in retinal degenerations [44], [45] and the rats were examined for the most important pathogens, however, none with relevance were detected. The light intensity was another factor that was closely watched since albino rats easily suffer retinal damages from increased light intensities. Therefore, the maximum light intensity for albino rat strains is recommended to be 60 lux (www.gv-solas.de/auss/hal/rattenhaltung.pdf). In the MedSchool the light intensity inside the cages ranged between 2 and 6 lux. In the VetSchool intensities of 6 to 28 lux were measured, with the exception of the top row of the female's rack where a light intensity of 130 lux was observed (Table 2). Furthermore, in the animal room of the VetSchool the illumination was unbalanced, as part of the room was brighter than the rest.

A correlation between the light intensity and severity of retinal degeneration was assumed as electroretinographical responses were slightly less in affected females than in males. However, functional or structural differences between animals kept in different rows and therefore at different light intensities were not observed. Since the impact of light intensities on the development of the retinal phenotype in LEW/Ztm-*Myo15^ci2^* rats remained unclear, this factor should be closely examined in further studies.

Based on these observations, we assume that the susceptibility to impaired vision is mediated by the interaction of the mutated *Myo15* gene, the genetic background and unknown environmental factors. The relevance of environmental factors for the development of retinal syndromes has been discussed for a long time. In the human Usher syndrome intrafamilial and interfamilial variations of retinal impairment have been reported, thereby indicating an involvement of genetic and non-genetic modifiers in disease expression [46]. A study by Liu et al. [47] indicates that even in humans clinical progression is influenced by environmental factors: monozygotic twins from an atypical USH family showed differences in the course of the disease and the severity of symptoms. Therefore, the varying phenotypes of humans, mice and rats harboring mutations in the C-terminal region of myosin XV might be due to different environmental conditions. Not only the retinal but also the vestibular phenotypes are absent in human patients.

Results of our study might provide insights on the question why the retinal phenotype in USH-patients and in USH-animal models follow such a variable course of disease. Our results suggest that retina degeneration manifests in a mutation-environment combination, most likely only affecting older animals. Therefore, to investigate the retinal phenotype in models of the human Usher syndrome we suggest using older animals housed in diverse environmental conditions.

In conclusion, the LEW/Ztm-*Myo15^ci2^* rat exhibits characteristic features of the human Usher syndrome. In contrast to most of established animal models for USH, the LEW/Ztm-*Myo15^ci2^* rat shows a retinal phenotype including photoreceptor degeneration, which is modified through currently unknown environmental factors. Consequently, this strain is an appropriate animal model for the investigation of the role of *Myo15* in human Usher syndrome, particularly for the investigation of the interaction between the mutation and environmental factors in modifying retinopathy.
